# Human Protoparvoviruses

**DOI:** 10.3390/v9110354

**Published:** 2017-11-22

**Authors:** Elina Väisänen, Yu Fu, Klaus Hedman, Maria Söderlund-Venermo

**Affiliations:** 1 Department of Virology, University of Helsinki, Helsinki 00290, Finland; elina.vaisanen@helsinki.fi (E.V.); yu.fu@helsinki.fi (Y.F.); klaus.hedman@helsinki.fi (K.H.); 2 Helsinki University Hospital, Helsinki 00290, Finland

**Keywords:** human protoparvovirus, bufavirus, tusavirus, cutavirus, discovery, epidemiology, gastroenteritis, cutaneous T-cell lymphoma, emerging viruses

## Abstract

Next-generation sequencing and metagenomics have revolutionized the discovery of novel viruses. In recent years, three novel protoparvoviruses have been discovered in fecal samples of humans: bufavirus (BuV) in 2012, tusavirus (TuV) in 2014, and cutavirus (CuV) in 2016. BuV has since been studied the most, disclosing three genotypes that also represent serotypes. Besides one nasal sample, BuV DNA has been found exclusively in diarrheal feces, but not in non-diarrheal feces, suggesting a causal relationship. According to both geno- and seroprevalences, BuV appears to be the most common of the three novel protoparvoviruses, whereas TuV DNA has been found in only a single fecal sample, with antibody detection being equally rare. Moreover, the TuV sequence is closer to those of non-human protoparvoviruses, and so the evidence of TuV being a human virus is thus far insufficient. Interestingly, besides in feces, CuV has also been detected in skin biopsies of patients with cutaneous T-cell lymphoma and a patient with melanoma, while all other skin samples have tested PCR negative. Even if preliminary disease associations exist, the full etiological roles of these viruses in human disease are yet to be resolved.

## 1. Introduction

Parvoviruses are small ssDNA viruses that infect a diverse group of animals; both vertebrates (subfamily *Parvovirinae*) and invertebrates (subfamily *Densovirinae*). Their non-enveloped capsid structure makes these viruses stable and very resistant to inactivation. Human parvoviruses are generally not easy to grow in standard cell culture systems, limiting the discovery of novel human parvoviruses in the past. However, in recent years great progress has been made in genetic sciences, where virion enrichment, host DNA depletion, sequence-independent amplification, next generation sequencing (NGS), and metagenomic analysis with diverse bioinformatic pipelines have revolutionized the discovery of novel viruses, including human parvoviruses.

For 30 years, human parvovirus B19 of the *Erythroparvovirus* genus was the only known indisputably human-pathogenic parvovirus, causing erythema infectiosum, arthropathies, anemias, and fetal death, while the highly prevalent adeno-associated viruses (AAV) of the *Dependoparvovirus* genus are apathogenic [1,2]. In 2005, two novel parvoviruses were discovered in humans: parvovirus 4 (PARV4) of the *Tetraparvovirus* genus in blood, and human bocavirus 1 (HBoV1) of the *Bocaparvovirus* genus in pediatric respiratory samples [3,4]. PARV4 is mainly detected in injecting drug users and hemophiliacs in the western world, but has not been associated with any specific symptoms, whereas HBoV1 causes mild to life-threatening respiratory tract infections in children [2]. Three more bocaviruses (HBoV2–4) have been discovered in stool, without clear clinical associations [5,6,7]. During the past six years, three novel parvoviruses have been described in humans, all belonging to the *Protoparvovirus* genus: bufavirus (BuV), tusavirus (TuV), and cutavirus (CuV) [8,9,10]. Although BuV has been associated with gastrointestinal symptoms and CuV is under study for its possible role in skin cancers, the etiological roles of these viruses in human disease are yet to be resolved. Here we review what is known about these new protoparvoviruses.

## 2. Bufavirus

### 2.1. Discovery and Taxonomy

**Figure 1:**
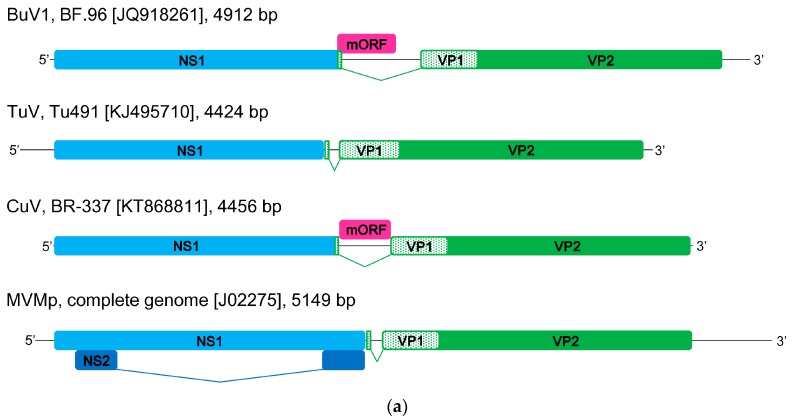

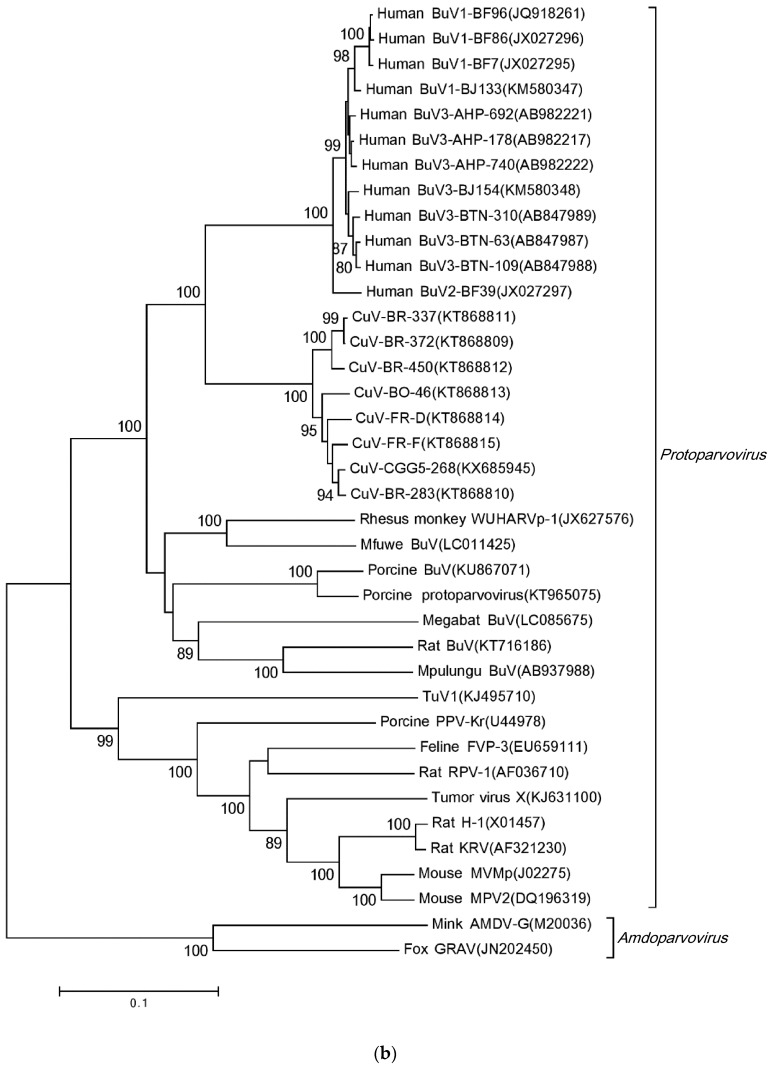

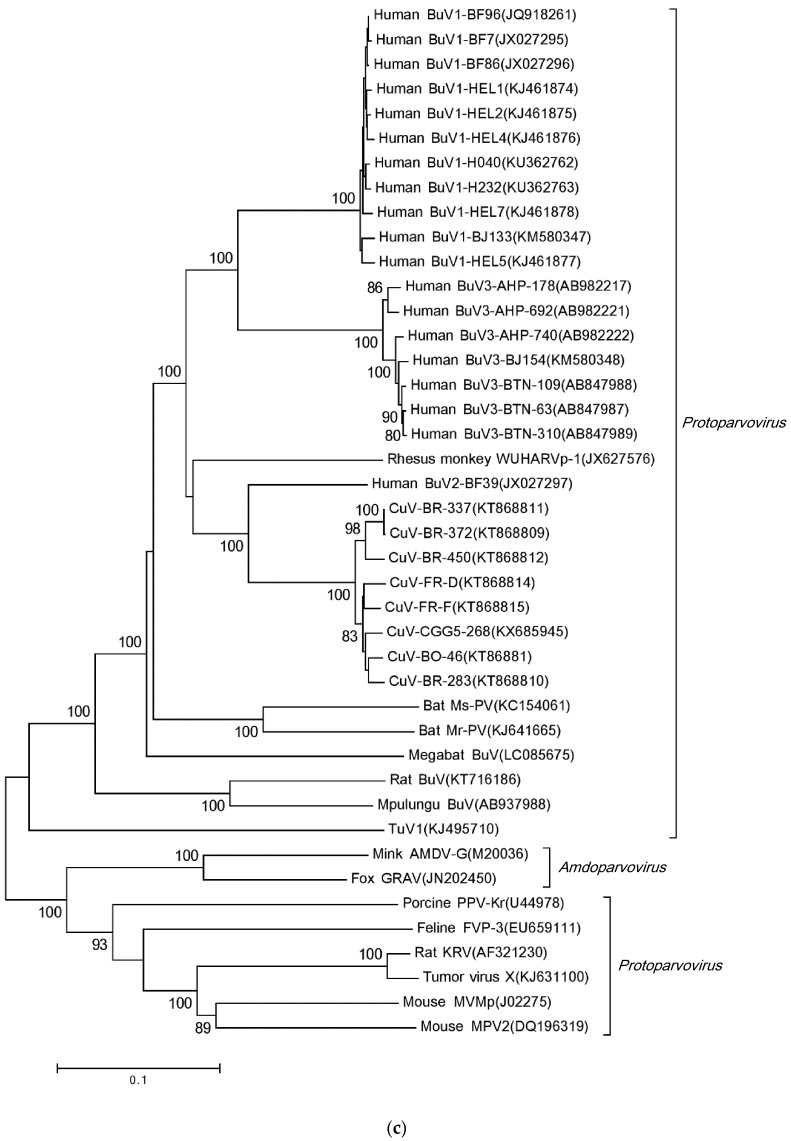

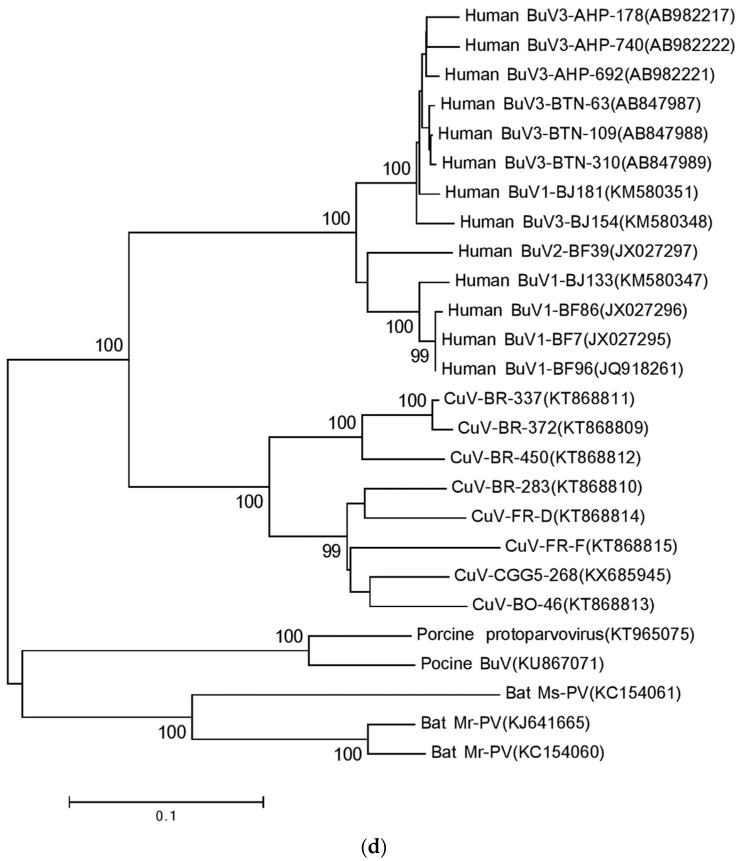
Schematic illustration of genomic structures and phylogenetic analyses of the sequences of BuVs, CuVs, TuV, and other members of *Protoparvovirus*. (**a**) Schematic illustration of BuV, TuV, CuV, and MVMp genomic structures. The hairpin regions are unknown for BuV, TuV, and CuV, and the hairpins of MVMp are represented as straight lines for this simplified scheme. Species, strain, GenBank no., and the length of genome are indicated; Phylogenetic analyses of (**b**) NS1 sequences; (**c**) VP2 sequences; (**d**) short middle ORF sequences (not available from TuV). The phylogenetic analysis was based on nucleic acid sequences. Sequences were aligned with ClustalX (Version 2.1) [14] using a protospacer adjacent motif (PAM) with a gap open penalty of 10 and gap extension penalty of 0.1. The neighbor-joining phylogenetic tree was generated with bootstrap values determined by 1000 replicates in the Molecular Evolutionary Genetics Analysis (MEGA)(Version 7.0) [15]. The evolutionary distances were calculated by the p-distance method. Bootstrap values are shown if >80%. NS1, non-structural protein 1; mORF, short middle ORF; VP1, viral capsid protein 1; VP2, viral capsid protein 2; NS2, non-structural protein 2.

Bufavirus was identified in 2012 in viral metagenomic analysis of fecal samples from diarrheic children in Burkina Faso [8]. The obtained sequence reads from one sample showed significant similarities to parvovirus sequences, and after filling the gaps between the NGS reads by PCR, a nearly full-length sequence of 4921 bp (GenBank # JQ918261) was constructed showing <39% and <31% identity of the NS1 (non-structural protein 1) and VP1 protein (viral capsid protein 1), respectively, to previously known protoparvoviruses. A unique middle open reading frame (ORF) of 130 amino acids was also identified that did not show similarity with any other parvovirus sequences (Figure 1a). Further PCR analysis of 98 rotavirus antigen-negative diarrheal fecal samples from Burkina Faso showed the presence of BuV DNA in 4/98 (4.1%) samples [8]. The complete protein-coding regions were determined for all BuV DNA isolates. Within the VP2 (viral capsid protein 2) region, one of the four isolates shared less than 73% amino-acid identity with the other isolates, whereas the non-structural NS1 regions were nearly identical. Subsequently, a second BuV genotype, bufavirus 2 (BuV2), was identified (GenBank # JX027297). The authors further studied 100 fecal samples from Chilean children with diarrhea and 63 samples from Tunisian children with non-polio acute flaccid paralysis, but only one BuV sequence was found among the latter [8].

Two years later, a third BuV genotype, BuV3 (GenBank # AB847987-9), was discovered in the diarrheal feces of 3/393 Bhutanese children [11]. All three current BuV genotypes contain an ATP- or GTP-binding Walker loop, phospholipase A_2_ (PLA2), two conserved replication initiator motifs, a glycine-rich sequence, and the same splice sites. Among the two major ORFs, the three genotypes are more divergent within the capsid proteins, sharing only 71–78% amino-acid identity within VP1 and 65–73% within VP2 [12]. In contrast, the NS1 regions of the three BuVs are nearly identical (94–96% amino acid identity). According to the International Committee on Taxonomy of Viruses (ICTV), parvoviruses in a single species encode NS1 proteins of >85% amino acid sequence identity [13]. Therefore, all three BuV genotypes can be phylogenetically classified into one species: *Primate protoparvovirus 1*, within the *Protoparvovirus* genus (Figure 1b).

### 2.2. PCR-Based Epidemiology

BuV DNA has been mainly searched for in feces, the sample type that the virus was originally identified in. BuV DNA has been detected in the diarrheal samples of children in Burkina Faso, Tunisia, Bhutan, Thailand, Turkey, China, and Finland, and of adults in Finland, the Netherlands, Thailand, and China (Table 1) [8,11,16,17,18,19,20,21,22]. In all studies, the BuV DNA prevalence in feces has been low, ranging from 0.3% to 4.1% (Table 1), and when reported, the viral titre was always low [16,22]. Recently, a partial sequence matching BuV3 was found by NGS in diarrheal feces from Peru in South America, expanding the geographic locations where BuV DNA has been detected (Table 1) [23]. Prevalence variation may occur even within one country; in China, the BuV DNA prevalence was 1.7% (9/520) among samples from the General Hospital of Beijing, whereas all samples (*n* = 1357) from the Children’s hospital of Chongqing were BuV DNA-negative [20]. So far, all BuV-DNA findings have been of genotypes 1 or 3, except the one original BuV2-DNA sequence from the single child in Burkina Faso [8,11,16,17,18,19,20,21,23], showing that BuV1 and 3 are geographically widespread.

In six studies, temporal clustering of BuV DNA detection to cold weather was observed as all or nearly all BuV DNA-positive samples were collected during September–April [11,18,19,20,21,22]. In four studies, negative controls were included; all fecal samples from non-diarrheic patients or healthy individuals (total *n* = 1855) were BuV DNA-negative (Table 1) [18,19,20,22]. However, in only two studies the non-diarrheal fecal samples were collected during the same time period as the BuV DNA-positive diarrheal samples [19,22]. This is of importance as in most studies BuV DNA was not detected throughout the year, and very often not at all during certain years.

Besides in feces, BuV DNA has been studied in nasal swabs of children and in cerebrospinal fluid (CSF) samples of adults and children [22,24]. Nasal swabs (*n* = 955) were collected in Finland from children with acute gastroenteritis (AGE, *n* = 172), acute respiratory tract infection (ARTI, *n* = 545), or both (*n* = 238), and BuV DNA was detected at low copy number in the nasal swab of one child with both AGE and ARTI (Table 1) [22]. However, the corresponding fecal sample of this child was BuV DNA-negative. In a study of central nervous system infections in Turkey, the CSF samples were collected from patients with a febrile disease and/or central nervous system infection with presumed viral etiology [24]. However, no BuV DNA sequences were detected by nested PCR in the CSF from these 93 Turkish children and 33 adults (Table 1).

### 2.3. Antibody-Based Epidemiology

A BuV enzyme immunoassay (EIA) has been developed to elucidate the prevalence of BuV1–3 IgG antibodies in various populations [22]. Humans have been shown to induce BuV IgG towards each of the three genotypes (Table 2). The BuV IgG prevalence was low in both Finnish-born adults (3.1%) and children (3.1%). Interestingly, among Asian-born university or hospital staff members working in Finland, BuV IgG was present in 5/12 of the tested subjects, suggesting that BuV is more prevalent in Asia [22]. The study moreover highlighted that the three BuV genotypes also represent serotypes, as no antibody cross-reactivity in EIA was observed. Further studies with larger cohorts from different parts of the world are ongoing to reveal the global BuV IgG prevalences in healthy populations and in different patient cohorts. In addition, no patients with serologically confirmed acute BuV infections have yet been reported. Such cases would be essential in order to broaden the knowledge of possible symptoms and diseases that BuV might cause.

### 2.4. Bufavirus-Like Animal Viruses

Since the discovery of human BuV, several animal species have been shown to have their own BuV-like viruses. The viruses phylogenetically closest to human BuVs have been detected in captive rhesus monkeys in the USA with a nucleotide identity of 77% in the *VP1* gene, and in wild baboons in Zambia (Figure 1) [25,26]. In both primate species, BuV-like viruses were detected either in the blood or in the spleen of the animal, indicating that these BuV-like viruses can cause systemic infections. Besides in primates, BuV-like viruses have so far also been identified in shrews, bats, rats, swine, and fur seals [12,26,27,28,29,30].

## 3. Tusavirus

Tusavirus, the name corresponding to Tunisian stool-associated parvovirus, was identified in 2014 using NGS and metagenomics in the fecal sample of an 18-month-old child with unexplained diarrhea in Tunisia [10]. Only one pool showed a single read of a parvovirus-like sequence, which was most similar to the NS1 sequence of a rat protoparvovirus. A near-complete genome sequence of 4424 bp was obtained (GenBank # KJ495710), including the two major ORFs, NS1 and VP1, typical of parvoviruses. Two conserved replication initiator sites and the Walker loop were also identified within NS1, as well as the PLA2 motif and an unusual serine-rich sequence near the VP1 N-terminus. TuV was shown to be quite distant from other protoparvoviruses: NS1 and VP1 showing the closest identities of 44% and 39%, respectively, to the Kilham rat parvovirus (Figure 1). TuV was thus proposed to be the prototype member for a new species, *Primate protoparvovirus 2*, in the *Protoparvovirus* genus [10]. However, the final classification is still pending due to the scarcity of this virus. No other TuV sequences were found in these Tunisian fecal samples by a nested PCR targeting the *NS1* gene, and thus the TuV DNA was present in only 1/180 (0.56%) diarrheal fecal samples. However, no other human-pathogenic viruses were detected in this sample.

No other studies of TuV DNA prevalence in any sample type have been reported, and only one other metagenomics study has disclosed sequences showing resemblance to TuV: a study of fur seals in Brazil described partial sequences ranging from 344 to 1519 nt in length, of both NS1 and VP regions with 39–82% similarity to TuV at the amino acid level [30]. Besides DNA, one serological study of TuV IgG in humans has been published, in which one child in Finland was shown by EIA to be barely positive for TuV IgG (1/228, 0.44%) [22]. 

## 4. Cutavirus

In 2016, yet another protoparvovirus, cutavirus, was detected by viral metagenomics in 2/245 diarrheal fecal samples from Brazilian children [9]. The near full-length 4456 bp sequence (GenBank # KT868811) of the CuV genome showed the typical major ORFs for NS1 and VP1 with 76% and 82% amino acid identity, respectively, to those of BuV2, the phylogenetically closest parvovirus, suggesting that CuV may be a distinct protoparvovirus species (Figure 1). The CuV *NS1* gene was, however, shorter than that of BuV; the alignment revealing an 11 amino-acid deletion near the C-terminus. The CuV genome was shown to contain the typical NS1 helicase motif with NTP-binding domains, as well as the VP1 PLA2 motif and glycine-rich regions. In addition, the sequence included a BuV-like middle ORF of unknown function, 20 amino acids shorter than that of BuV, with a 45% identity (Figure 1).

By CuV VP-specific nested PCR, three more CuV sequences were detected, 2 more in Brazilian fecal samples and one in one hundred fecal samples from Botswana. In all, the CuV DNA fecal prevalence was 4/245, 1.6%, in Brazilian and 1/100, 1.0%, in Botswanan diarrheic children [9]. These CuV genomes were only partly sequenced. To search for other pathogens in the five CuV DNA-positive samples, NGS was performed on the individual samples. In one of the Brazilian samples, no other human viruses were detected [9]. In the other three Brazilian samples, one contained rotavirus A and AAV, one astrovirus and adenovirus, and one rotavirus A and anellovirus. The Botswanan sample harbored both picobirna- and anellovirus.

Interestingly, by further in silico screening of existing NGS libraries, CuV DNA was retrospectively detected in two skin biopsies of French cutaneous T-cell lymphoma (CTCL) patients, thereby resulting in the virus name [9]. By CuV-nested PCR, 15 additional CTCL skin samples were screened, of which two more (2/15, 13.3%) were found to be CuV DNA-positive. Skin samples of other cancers (*n* = 10) and non-malignant skin biopsies (*n* = 19) were all CuV PCR-negative (Table 3). Later on, CuV DNA was detected with NGS in the malignant skin lesions of one melanoma patient in Denmark [31], while 9 additional melanoma samples were CuV DNA-negative when using both NGS and PCR. The CuV nt sequence from Denmark was 96.5–93.7% identical to the previously published three sequences covering the entire NS1 and VP coding regions (Table 4, data calculated for this review, and Figure 1). When comparing all known CuV nt sequences (of 3980 nt in length), the BR-283 sequence from Brazilian feces was closer to the Danish and French sequences from skin than to the other Brazilian sequences (Table 4). These data indicate that the sequences of CuV strains found in skin and feces are similar.

## 5. Discussion

Three protoparvoviruses, BuV, TuV and CuV, are the newest parvoviruses identified among humans [8,9,10]. All three were originally discovered in diarrheal feces of children by using virion enrichment and nuclease treatment prior to NGS, suggesting that the discovered viral DNA in the samples might have been protected by a capsid structure. No cell culture experiments have been done. The subsequent studies have instead focused on finding the DNAs of these viruses, or antibodies towards them, in human samples. As parvoviruses generally infect host-specifically and human parvoviruses grow poorly in cell cultures, DNA and antibody detection are the most feasible ways to establish these viruses as human viruses. Studies and findings on TuV DNA or antibodies are, however, scarce; only one DNA isolate exists and only one child has been barely TuV IgG-positive [10,22]. Whether TuV truly is a human parvovirus and not of, e.g., dietary origin, requires further investigation.

Of these three protoparvoviruses, BuV was discovered first and has been studied the most. The fact that BuV DNA is found near-exclusively in the feces of patients with diarrhea, often without other pathogen coinfections, whereas non-diarrheal fecal samples all have been BuV DNA-negative, suggests that BuV might be causative for gastroenteritis. However, the low viral loads and infrequent detection of BuV DNA point to a less prominent role in the overall burden of gastroenteritis. Whether BuV is involved in other clinical manifestations is not yet known, as acute primary BuV infections have not yet been serologically identified. The presence of IgG in humans, however, indicates that BuV is a true, capsid-covered infectious virus. The remarkable difference in BuV seroprevalence between Finland and Asia is interesting and under ongoing investigation. The reasons for such diversity might be cultural, genetic, or demo-geographic.

The newest member of the human protoparvoviruses is CuV, the DNA of which has been detected both in the diarrheal feces of children and in 4/17 skin samples of patients with CTCL (mycosis fungoides), whereas all 31 non-CTCL skin biopsies were PCR negative [9]. In addition, one Danish melanoma skin biopsy was found to be CuV DNA-positive. That the virus has been detected both in feces and skin suggests that CuV is a human virus and may cause systemic infection. Further PCR and sero-epidemiological studies are ongoing to assess the cancer association and the global distribution of this novel human virus.

In conclusion, according to DNA and antibody data, BuV and CuV are indeed human viruses, whereas for TuV the evidence is inconclusive. Further DNA studies of diverse sample materials combined with serological analyses are needed to establish the pathogenicity, or the absence of it, regarding, in particular, gastrointestinal and neoplastic illnesses.

## Figures and Tables

**Table 1:** BuV DNA studies of subjects with or without diarrhea.

Study	Sample Type	Country	Age (Range) If Known	Sampling Time	*n*	Positive (%)	Symptoms	Genotype
[8]	Feces	Burkina Faso	<5 yr	November 2008–February 2010	98	4 (4.1%)	GE	Three BuV1, one BuV2
	Feces	Tunisia	“children”	NA	63	1 (1.6%)	NPAFP	unknown
	Feces	Chile	“children”	NA	100	0 (0.0%)	GE	-
[11]	Feces	Bhutan	<5 yr	February 2010–January 2012	393	3 (0.8%)	GE	All BuV3
[16]	Feces	Finland	median 51.5 yr (0–99 yr)	October 2012–March 2013; April–May 2013	629	7 (1.1%)	GE	Six BuV1, one unknown
[17]	Feces	The Netherlands	median 47 yr (0–97 yr)	2005–2009	27	1 (3.7%)	GE	BuV3
[19]	Feces	Thailand	median NA (0–97 yr)	January 2009–April 2014	1495	4 (0.3%)	GE	All BuV1
	Feces	Thailand	median NA (0–39 yr)	February 2010–July 2014	**741**	0	HFMD	-
[18]	Feces	Turkey	mean 19.5 mo (1–60 mo)	September 2004–June 2011	583	8 (1.4%)	GE	Six BuV3, two unknown
	Feces	Turkey	mean 17.3 mo (NA), age matched	February–September 2013	**148**	0	healthy	-
[20]	Feces	China (General hospital, Beijing)	median 35 yr (1 mo–85 yr)	2010–2014	520	9 (1.7%)	GE	Four BuV1, five BuV3
			“children”	NA	**76**	0	non-GE	-
	Feces	China (Children’s hospital, Chongqing)	median 10 mo (1 day–14 yr)	2010–2013	1357	0	GE	-
			“children”	NA	**345**	0	non-GE	-
[21]	Feces	Tunisia	median 7.0 mo (0.5–60 mo)	October 2010–March 2012	203	2 (1.0%)	GE	Both BuV1
[22]	Feces	Finland	median 14 mo (6 days–15.6 yr)	September 2009–August 2011	172	2 (1.2%)	GE	Unknown ^1^
					**545**	0	ARTI	-
					238	1 (0.4%)	GE & ARTI	Unknown ^1^
	nasal swab	Finland	median 14 mo (6 days–15.6 yr)	September 2009–August 2011	172	0	GE	-
					545	0	ARTI	-
					238	1 (0.4%)	GE & ARTI	Unknown ^1^
[24]	CSF	Turkey	median 32 yr (0–96 yr)	October 2011–April 2015	126	0	febrile illness and/or CNS infection	-
[23]	Feces	Peru	Unknown	NA	300	Unknown ^2^	GE	BuV3

^1^ Short sequence only from conserved NS1 region, cannot separate genotypes; ^2^ by NGS, only the presence of BuV3 sequence was reported, prevalence cannot be calculated; ARTI, acute respiratory tract infection; CNS, central nervous system; CSF, cerebrospinal fluid; GE, gastroenteritis; HFMD, hand, foot, and mouth disease; mo, month(s); NA, not available; non-GE, patients who had sought medical care from the same hospitals for reasons other than GE; NPAFP, non-polio acute flaccid paralysis; yr, year(s); *n* of the non-diarrheic control patients are bolded.

**Table 2:** BuV IgG-positive adults and children. The origins of the subjects are shown in the header.

Genotype	Adults				Children
	Asia, *n* = 12			Finland, *n* = 163	Finland, *n* = 228
	Middle East	India	China		
BuV1	1 ^1^	3		1	2 ^2^
BuV2	1 ^1^		1	4	4 ^2^
BuV3	1 ^1^				2
Any BuV	1 ^1^	3	1	5	7 ^2^

^1^ One individual was positive for all three BuVs; ^2^ one individual was positive for both BuV1 and BuV2; Any BuV, sums of the BuV IgG-positive individuals.

**Table 3:** CuV DNA prevalences in fecal and skin samples.

Study	Sample Type	Country	*n*	Positive (%)	Symptoms/Diagnosis
[9]	Feces	Brazil	245	4 (1.6%)	Diarrhea
[9]	Feces	Botswana	100	1 (1.0%)	Diarrhea
[9]	Skin	France ^1^	NA	2	CTCL, Mycosis fungoides
[9]	Skin	France	15	2 (13.3%)	CTCL, Mycosis fungoides
[9]	Skin	France	10	0 (0%)	Skin carcinoma
[9]	Skin	France ^1^	NA	0	Parapsoriasis
[9]	Skin	2 France; 6 NA	8	0 (0%)	Parapsoriasis
[9]	Skin	NA	8	0 (0%)	Eczema or eczematoid dermatitis
[9]	Skin	NA	3	0 (0%)	Healthy
[31]	Skin	Denmark	10	1 (10%)	Melanoma

^1^ 2 CuV DNA-positive skin samples from CTCL patients were detected with NGS and PCR. However, it is not known how many CTCL skin samples were tested by NGS, whereby these 2 cases are not included in the overall prevalence. The same holds for the 2 CuV DNA-negative parapsoriasis skin samples tested in NGS pools. NA, not available; CTCL, cutaneous T-cell lymphoma.

**Table 4:** Nucleotide identities between the currently available CuV sequences. A sequence of approximately 3980 nts, covering the partial NS1 and complete VP regions (nts 474–4456 of BR-337 [KT868811]) was used for calculations. Strain, sample type, and GenBank no. indicated.

	% Nucleotide Identity							
	BR-337, Feces [KT868811]	BR-283, Feces [KT868810]	BR-372, Feces [KT868809]	BR-450, Feces [KT868812]	BO-46, Feces [KT868813]	FR-D, Skin [KT868813]	FR-F, Skin [KT868813]	CGG5-268, Skin [KX685945]
BR-337, feces [KT868811]	100.0							
BR-283, feces [KT868810]	94.4	100.0						
BR-372, feces [KT868809]	99.5	94.6	100.0					
BR-450, feces [KT868812]	96.9	94.4	97.1	100.0				
BO-46, feces [KT868813]	93.9	96.0	94.1	93.9	100.0			
FR-D, skin [KT868813]	93.8	96.2	93.8	93.9	95.0	100.0		
FR-F, skin [KT868813]	94.4	96.6	94.5	94.5	95.5	95.9	100.0	
CGG5-268, skin [KX685945]	94.1	97.1	94.2	94.2	96.0	96.1	96.7	100.0
